# Peripheral Administration of the Kv1.3-Blocking Peptide HsTX1[R14A] Improves Cognitive Performance in Senescence Accelerated SAMP8 Mice

**DOI:** 10.1007/s13311-023-01387-z

**Published:** 2023-05-24

**Authors:** Yijun Pan, Yoshiteru Kagawa, Jiaqi Sun, Deanna S. Deveson Lucas, Ryusuke Takechi, John C. L. Mamo, Dorothy C. C. Wai, Raymond S. Norton, Liang Jin, Joseph A. Nicolazzo

**Affiliations:** 1 Drug Delivery, Disposition and Dynamics, Monash Institute of Pharmaceutical Sciences, Monash University, Parkville, VIC 3052 Australia; 2 Florey Institute of Neuroscience and Mental Health, University of Melbourne, Parkville, VIC 3052 Australia; 3 Department of Organ Anatomy, Graduate School of Medicine, Tohoku University, Sendai, Miyagi 980-8575 Japan; 4 Monash Bioinformatics Platform, Biomedicine Discovery Institute, Monash University, Clayton, VIC 3800 Australia; 5 School of Biomedical Sciences, Curtin University, Bentley, WA 6102 Australia; 6 School of Public Health, Curtin University, Bentley, WA 6102 Australia; 7 Medicinal Chemistry, Monash Institute of Pharmaceutical Sciences, Monash University, Parkville, VIC 3052 Australia; 8 ARC Centre for Fragment-Based Design, Monash University, Parkville, VIC 3052 Australia

**Keywords:** HsTX1[R14A], Kv1.3 channels, Neuroinflammation, SAMP8, Behavioral assessment, Transcriptomics

## Abstract

Increased expression of the voltage-gated potassium channel Kv1.3 in activated microglia, and the subsequent release of pro-inflammatory mediators, are closely associated with the progression of Alzheimer’s disease (AD). Studies have shown that reducing neuroinflammation through the non-selective blockade of microglial Kv1.3 has the potential to improve cognitive function in mouse models of familial AD. We have previously demonstrated that a potent and highly-selective peptide blocker of Kv1.3, HsTX1[R14A], not only entered the brain parenchyma after peripheral administration in a lipopolysaccharide (LPS)-induced mouse model of inflammation, but also significantly reduced pro-inflammatory mediator release from activated microglia. In this study, we show that microglial expression of Kv1.3 is increased in senescence accelerated mice (SAMP8), an animal model of sporadic AD, and that subcutaneous dosing of HsTX1[R14A] (1 mg/kg) every other day for 8 weeks provided a robust improvement in cognitive deficits in SAMP8 mice. The effect of HsTX1[R14A] on the whole brain was assessed using transcriptomics, which revealed that the expression of genes associated with inflammation, neuron differentiation, synapse function, learning and memory were altered by HsTX1[R14A] treatment. Further study is required to investigate whether these changes are downstream effects of microglial Kv1.3 blockade or a result of alternative mechanisms, including any potential effect of Kv1.3 blockade on other brain cell types. Nonetheless, these results collectively demonstrate the cognitive benefits of Kv1.3 blockade with HsTX1[R14A] in a mouse model of sporadic AD, demonstrating its potential as a therapeutic candidate for this neurodegenerative disease.

**Supplementary Information:**

The online version contains supplementary material available at 10.1007/s13311-023-01387-z.

## Introduction

There is growing evidence for a prominent role of neuroinflammation in the pathogenesis of Alzheimer’s disease (AD), along with β-amyloid (Aβ) and tau pathology [1–3]. Chronic neuroinflammation is attributed to activation of microglia, the resident immune cells of the central nervous system (CNS) [4, 5]. In AD, microglia present a phenotype characterized by increased pro-inflammatory cytokine production, impaired phagocytosis, and deficient Aβ debris clearance [6]. While non-specific suppression of microglia shows promise in improving cognitive function in mouse models of AD, these approaches may cause undesirable effects such as reducing phagocytic activity of microglia, potentially exacerbating Aβ accumulation. Therefore, it is highly desirable to identify therapeutic targets that can selectively suppress the activated state of pro-inflammatory microglia.

One such target is the voltage-gated potassium channel Kv1.3, a member of the family of transmembrane proteins regulating cell membrane potential [7, 8]. Kv1.3 channels help to maintain a negative membrane potential, which provides the driving force for Ca^2+^ entry through store-operated inward-rectifier calcium channels necessary for subsequent cytokine production by microglia [9]. Kv1.3 channels are selectively upregulated in pro-inflammatory microglia, leading to excessive release of cytokines that contribute to inflammatory cascades [10]. Recent studies have revealed enhanced levels of microglial Kv1.3 in 5 × FAD mice (a model of familial AD that rapidly develops severe Aβ pathology) and in brains from individuals with AD [11, 12]. A Kv1.3 blocker PAP-1 (IC_50_ = 2 nM) [13] reduced microglia-mediated neuroinflammation in 5 × FAD and APP/PS1 mice (another model of familial AD), and inhibited microglia-mediated neurotoxicity in 5 × FAD mice [7, 14, 15] and in APP/PS1 mice [12]. Interestingly, blocking Kv1.3 also enhanced the ability of microglia to phagocytose Aβ in APP/PS1 mice [12], and enhanced the clearance of Aβ from the brain in 5 × FAD mice [14, 15]. Thus, Kv1.3 is a promising target for selective suppression of the pro-inflammatory activities of microglia in AD. Although Kv1.3 channel blockers have been studied primarily for their potential to modulate microglia-mediated neuroinflammation, it is important to note that Kv1.3 channels are also expressed in other cell types within the brain, including neurons and neural progenitor cells [16]. It has been demonstrated that genetic silencing of neuronal Kv1.3 reduces neuronal death [17], and pharmacological blockade of Kv1.3 channels on neural progenitor cells promotes their proliferation [18]. Therefore, blockade of Kv1.3 on these cells may promote neurogenesis, thereby providing benefit to brain health [19], in addition to the benefits of blockade of microglial Kv1.3.

Given the non-selective nature of Kv1.3 blockers such as PAP-1, development of Kv1.3 blockers with higher selectivity is essential for realising the therapeutic potential of Kv1.3 blockade in AD. Furthermore, it should be noted that the transgenic mouse models previously used to demonstrate the disease-modifying effects of Kv1.3 blockade (i.e. APP/PS1 and 5 × FAD mice) do not reflect the majority of clinical AD cases, i.e. sporadic AD, that occur in elderly humans (without excessively elevated, lifelong cerebral amyloid precursor protein (APP) expression or Aβ production) [20]. Therefore, to enhance the translational potential of Kv1.3 blockers in AD, their effectiveness in a mouse model of sporadic AD needs to be assessed.

We have developed and characterized a Kv1.3 peptide-based blocker, HsTX1[R14A], that is stable and has a high affinity for Kv1.3 (IC_50_ = 45 pM) and a 2000-fold selectivity for Kv1.3 over other Kv channels [21, 22]. Our work has demonstrated that HsTX1[R14A] effectively inhibits microglia-mediated neurotoxicity by attenuating the release of pro-inflammatory mediators such as tumor necrosis factor-alpha (TNF-α) and nitric oxide from lipopolysaccharide (LPS)-activated microglia in vitro [23]. Following peripheral administration in a mouse model of neuroinflammation induced by LPS, HsTX1[R14A] readily crosses the blood–brain barrier (BBB) [24] to achieve microglial Kv1.3 blockade, with a substantial reduction in plasma and brain levels of cytokines, including TNF-α, interleukin 1β (IL-1β) and interleukin-6 (IL-6) [23, 25]. It should be noted that the reduction of cytokines observed in the brain could be a combined effect of HsTX1[R14A] acting on both microglia in the brain and peripheral immune cells. Nevertheless, our studies suggest that HsTX1[R14A] may have beneficial effects in diseases with a significant Kv1.3-mediated neuroinflammatory component, such as AD.

We therefore evaluated the beneficial effects of peripherally-administered HsTX1[R14A] in senescence accelerated murine prone 8 (SAMP8) mice, which exhibit memory deficits and cognitive decline in the absence of APP overexpression [26], thus closely mimicking what is observed in sporadic AD. We first compared Kv1.3 mRNA expression in microglia isolated from SAMP8 mice and senescence-accelerated mouse resistant strain 1 (SAMR1) mice, which serve as a control to SAMP8 mice, at four months of age, an age when AD pathological changes begin to emerge [27]. We then assessed the impact of peripherally-administered HsTX1[R14A] (8-week dosing) on cognition and the brain transcriptome in SAMP8 mice. This study provides proof-of-concept data to support HsTX1[R14A] as a new Kv1.3 blocker to improve cognition and brain health in a mouse model of sporadic AD.

## Methods

### Materials

HsTX1[R14A] was purchased from Peptides International (Louisville, KY). RNeasy Lipid Tissue Mini Kit and RNeasy Micro Kit were purchased from Qiagen (Hilden, Germany). MACS^®^ CD11b MicroBead and Adult Brain Dissociation Kit were purchased from MiltenyiBiotec (North Rhine-Westphalia, Germany). Phosphate-buffered saline (PBS) and Taqman™ primers were sourced from ThermoFisher Scientific (Waltham, MA). iScript™ Reverse Transcription Supermix was purchased from Bio-Rad (Hercules, CA).

### Animals

Animal experiments were approved by the Monash Institute of Pharmaceutical Sciences (MIPS) Animal Ethics Committee (MIPS.26951) and performed in accordance with the National Health and Medical Research Council guidelines for the care and use of animals for scientific purposes. Equal numbers of male and female mice were used in this study. SAMP8 is a naturally occurring mouse line that displays a phenotype mimicking AD in humans, and SAMR1 mice are routinely used as controls to SAMP8 mice [26, 27]. Male and female SAMP8 and SAMR1 mice were bred at the Animal Resources Centre (Canning Vale, Western Australia, Australia), transferred to MIPS (Parkville, Victoria, Australia) at 5–6 weeks old, and thereafter housed in a 12:12 reversed cycle holding room (lights on at 7 PM) with ad libitum access to standard rodent chow and water until the terminal experiments.

### Isolation of Microglia from SAMR1 and SAMP8 Mice to Quantify Target Genes

It has been reported that AD pathological changes begin to emerge in 4-month-old SAMP8 mice [27]; however, whether Kv1.3 and pro-inflammatory markers are upregulated at the mRNA level in SAMP8 microglia compared to SAMR1 microglia at this age is yet to be confirmed. Confirming the upregulation of Kv1.3 in SAMP8 mice would rationalize the use of HsTX1[R14A] (a Kv1.3 blocker) to modify the disease pathology in this AD mouse model at 4 months of age.

Mice were euthanized via cervical dislocation under isoflurane anesthesia. The brains were removed immediately for microglia isolation using MACS^®^ MicroBead Technology (CD11b), in combination with the Adult Brain Dissociation Kit as described previously [23]. Total RNA was isolated from microglia isolated from 4-month-old SAMR1 (n = 3) and SAMP8 (n = 4) mice using the RNeasy Micro Kit as per the manufacturer's protocol. The mRNA targets were Kcna3 (Kv1.3), pro-inflammatory cytokines IL-1β and IL-6, a pro-inflammatory microglia marker (prostaglandin-endoperoxide synthase 2, Ptgs2), and an anti-inflammatory microglia marker (Arginase 1, Arg1). For PCR, iScript™ Reverse Transcription Supermix for RT-qPCR was used; thermocycling and the measurement of mRNA expression by quantitative analysis were carried out in a CFX96 system (Bio-Rad, Hercules, CA). The threshold cycles (Ct) were calculated automatically using the CFX manager software. To determine relative mRNA expression between microglia isolated from SAMR1 and SAMP8 mice, the fold-change method (2^–ΔΔCt^) was employed [28], with *Gapdh* used as a housekeeping gene.

### HsTX1[R14A] Dosing

Dosing was initiated at 4 months of age (the age when microglial Kv1.3 is upregulated in SAMP8 mice as assessed by PCR). Only SAMP8 mice were used for dosing, as the purpose of this study was to demonstrate if HsTX1[R14A] could improve cognitive function in SAMP8 mice by modulating microglia-mediated neuroinflammation at an early disease stage. All mice were housed as a group of 3–4 with cage enrichment; in each cage, mice were randomized to subcutaneous dosing every other day with either HsTX1[R14A] (1 mg/kg in PBS) or PBS at ~8 PM (1 h after the light phase started). Injection volumes of each administered dose were adjusted according to the body weight of each mouse (which was assessed weekly) to ensure dose consistency across the cohort.

### Behavioral Assessment

**Fig. 1:**
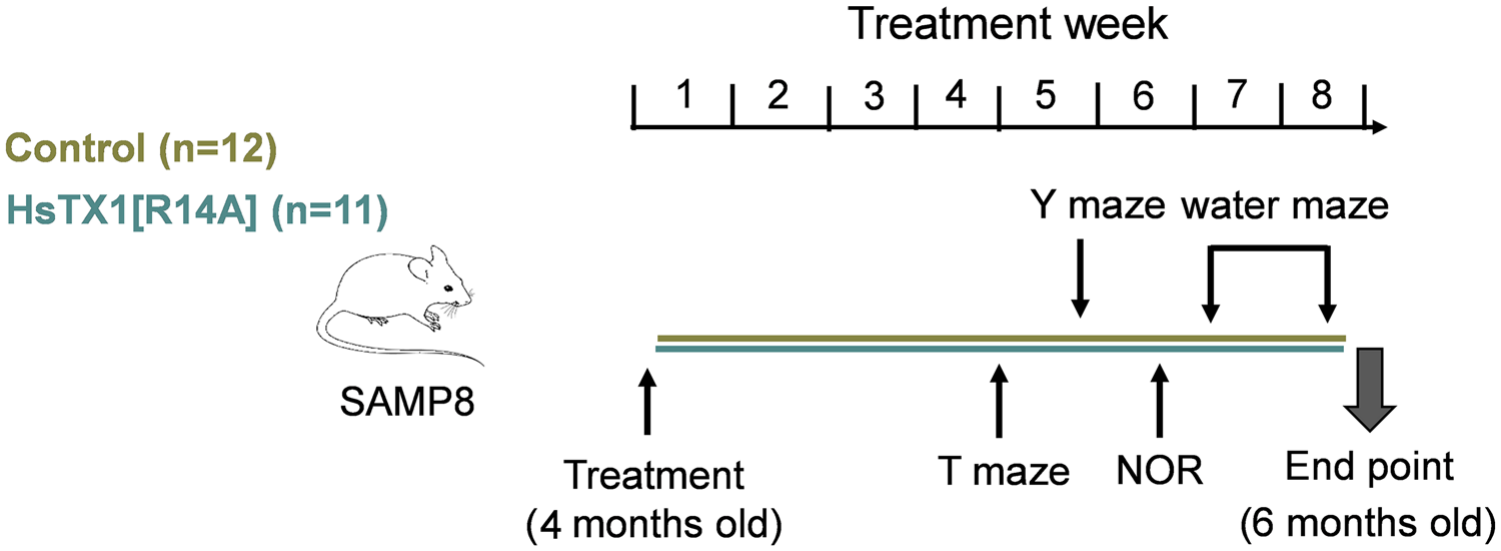
Flow chart depicting the subcutaneous dosing regimen of HsTX1[R14A] (1 mg/kg) or vehicle in SAMP8 mice and the subsequent behavioral assessment and end point studies. The behavioral assessments were spread across the last 4 weeks of treatment to ensure animal welfare and to prevent stress-induced behavioral changes from intensive testing in a short period. End point experiments were performed immediately after the 2-month treatment

All mice were assessed by the T maze spontaneous alternation, Y maze, novel object recognition (NOR) and water maze tests to assess working memory, short-term spatial learning and memory, episodic memory, and long-term memory, respectively. These tests were also utilized to assess the locomotion and anxiety levels of the mice. The sequence of the behavioral assessments is summarized in Fig. 1. The behavioral tests were performed over the last 4 weeks of dosing (i.e. one test per week). There was at least a 12-h gap between dosing and behavioral assessment. All videos were analyzed using Viewer tracking software (Biobserve GmbH, Sankt Augustin, Germany) and object exploration time in the NOR test was verified by manual counting performed by Y.P. and J.S. independently. The mice were acclimatized for 60 min in the experimental room prior to all behavioral assessments.

*T maze spontaneous alternation* was performed as described previously to assess working memory [29]. The T-maze apparatus has three equal arms in the shape of a capital T (30 cm L × 10 cm W × 20 cm H). The mice were introduced to the T maze at the base of a pseudorandomized arm and allowed to move freely through the maze for 3 min. The movement of the animal in the maze was recorded by a roof camera, and the sequence of arm entries was recorded (arm entry was counted if both the head and the base of the tail entered the arm). A correct alternation was defined as a non-repeated entry into the arms for three consecutive entries. The percentage of spontaneous alternations was calculated as described previously [29]. The maze was cleaned between sessions using ethanol to remove any olfactory cues that could potentially affect the behavior of the next mouse being assessed.

*The Y maze* was performed to assess short-term spatial learning and memory. The maze consists of three arms (50 cm L × 16 cm W × 31 cm H) in the shape of a capital Y with spatial cues on the internal wall of each arm. For training, mice were introduced into the Y maze with one of the arms blocked, and allowed to explore the maze for 3 min. After a 3–3.5 h retention interval, the mice were re-introduced into the maze but with access to all three arms, and allowed to explore the maze for 3 min. The previously-blocked arm is referred to as the ‘novel arm’, the arm from which mice were introduced into the maze is referred to as the ‘start arm’, and the other arm is the ‘familiar arm’. The allocation of these arms to the mice was pseudorandomized. The time mice spent in each arm was analyzed by Viewer tracking software. Mice with intact spatial learning and memory are expected to spend a significantly longer time in the novel arm than the familiar arm. The distance traveled by the mice during the training session was used to measure locomotor activity.

*The NOR *task was performed to assess episodic memory. On day 1, mice were allowed to acclimatize to an open arena (40 cm L × 40 cm W × 30 cm H) for 5 min, and the distance traveled by mice was analyzed using Viewer tracking software to assess locomotor activity and to monitor if mice were acclimatized to the open arena. On day 2, mice were re-introduced to the arena for 5 min for acclimatization. Immediately after acclimatization, two identical objects were placed into the arena, and mice were allowed to explore the arena for 10 min (i.e. training session). Mice were then returned to their home cage for resting. Mice that exhibited right/left object bias (i.e. spending > 60% of time exploring either one object) in the training session were removed from the study. After 3–3.5 h from the training session, the mice were re-introduced to the arena with one of the familiar objects (pseudorandomized) replaced with a novel object. The time that mice spent exploring each object (classified by nose sniffing the object) on day 2 was determined by Viewer tracking software for the calculation of discrimination index [30]. The object exploring time was also verified via manual counting (performed independently by Y.P and J.S.). Mice that could recognize the novel object were expected to give a positive discrimination index [30].

*The water maze* was employed to assess spatial learning and long-term spatial memory of mice. As described previously [30], mice were introduced into a circular pool (120 cm in diameter and 20 cm in depth) filled with water colored by nontoxic black paint. The pool was equipped with four internal cues (corresponding to the compass locations of north, south, east and west), dividing the pool into four quadrants (target, right, opposite, left in clockwise orientation) and external cues were placed around the room. Water temperature was maintained at 24 ± 2 °C using an in-built heating system. Mice were placed into the pool at the same position for each trial. On day 1, the latency to swim to a circular escape platform (12 cm in diameter, placed in the target quadrant) submerged 1 cm below the water surface was assessed across four trials (D1T1, D1T2, D1T3, D1T4 where D refers to day and T refers to training session) administered at 30 ± 10 min intervals. A visual cue (10 cm in height) was attached to the platform to allow the mice to easily navigate to the platform. Each trial was ≤ 90 s in duration, and mice that did not reach the platform within this 90 s period were guided onto the platform and allowed to sit on it for 15 s before being returned to the home cage. With repetitive training, the latency of the mice to travel to the escape platform is expected to decrease. On day 2, mice were again placed into the pool, but without visual cues attached to the escape platform. Three spatial trials (D2V1, D2V2, D2V3 where D refers to day and V refers to visual trial session) were held at 30 ± 10 min intervals, where the latency to the platform was recorded. All mouse behaviors were recorded via a roof camera positioned above the pool and the data were extracted using Biobserve Viewer with water maze plugin.

### Organ Harvesting

At the end of the behavioral assessment, mice were perfused with ice-cold PBS transcardiacally under anaesthesia. The brain, liver, and kidney were carefully removed and weighed to assess the impact of HsTX1[R14A] on these organs. The brain weight was assessed as this value could be an indicator of cognitive capacity of the mice [31]. Weights of livers and kidneys were also measured to ensure that there was no peptide-induced enlargement or shrinkage of these important peripheral organs from the chronic administration of HsTX1[R14A] [32]. All collected organs were snap frozen and stored at – 80 ^ο^C until analysis.

### Transcriptomic Analysis of the Impact of HsTX1[R14A] on SAMP8 Mouse Brains

We performed transcriptomics on the whole brain RNA to explore biological process, molecular function, cellular component and gene sets that may be affected by HsTX1[R14A] (not just focusing on its impact on microglia-mediated inflammation) and to evaluate the impact of HsTX1[R14A] on brain health. Total RNA from the brains of SAMP8 mice treated with HsTX1[R14A] or PBS for 8 weeks was extracted using RNeasy Lipid Tissue Mini Kit (Qiagen). All samples (2 µL) were measured using the Invitrogen Qubit™ and assayed with the RNA HS assay (ThermoFisher, Waltham, MA). Sample were sized and measured for RNA integrity using the Agilent Bioanalyzer 2100 and RNA Nano Assay Kit (Santa Clara, CA), according to the manufacturer's instructions. The samples were processed using an MGI RNA Directional Library Preparation Set V2 (MGI Tech, Shenzhen, China), according to the manufacturer's instructions, with the following modifications/options: (1) RNA was fragmented at 87 °C for 6 min to target insert size of 200-400 bp; (2) adapters were diluted 1 in 5; and (3) the libraries were amplified with 13 cycles of PCR. The libraries were pooled in equimolar concentrations and sequenced using an MGI DNBSEQ-2000RS with reagent chemistry V3.1 (MGI Tech). Fastq files were processed using the Laxy tool [33] and the NfCore/RNAseq (v3.2) pipeline [34]. Reads were aligned to the *Mus musculus* GRCm38 reference using STAR aligner [35] and quantified using Salmon [36] producing the raw genes count matrix. Various quality control metrics were generated and summarized in a multiQC report [37]. The raw counts were analysed with Degust [38], a web tool that performs normalization using the trimmed mean of M values (TMM) [39], producing counts per million (CPM) library size normalisation [39] and differential expression analysis using limma/voom [40]. Male and female mouse brain samples were combined for each treatment. All samples were then ‘batch corrected’ for sex to remove the effect of sex chromosome expression on the dataset. Statistically significant, differentially-expressed genes were defined as those with a False Discovery Rate (FDR) ≤ 0.05, and a log_2_Fold change (log_2_FC) ≥ $\pm$ 1. Functional enrichment analysis was performed using STRING Version 11.5 V (https://string-db.org/), and R studio (Boston, MA) was used for graphing using ggplot [41]. Gene Set Enrichment Analysis (GSEA) was performed with the software provided by the Massachusetts Institute of Technology [42, 43] on pre-ranked data (using the limma t.stat function) with all the observed genes in the RNA-Seq dataset. Normalized enrichment score (NES) and FDR were used to quantify enrichment magnitude and statistical significance, respectively, comparing PBS- and HsTX1[R14A]-treated mouse brains, using the GSEA hallmark and m2 curated gene sets. The RNA-seq data have been deposited in the NCBI Gene Expression Omnibus with accession number GSE221352 (https://www.ncbi.nlm.nih.gov/geo/query/acc.cgi?acc=GSE221352).

### Statistical Analysis

Results are expressed as mean ± SEM unless stated otherwise. Student’s t-tests and analysis of variance (ANOVA) tests were performed where appropriate for mRNA expression and behavioral assessments. Data analysis and graphing were performed using GraphPad version 9.3.1 (Prism, San Diego, CA).

## Results

### Levels of Genes Associated with Microglial Pro-Inflammatory Activation are Elevated in 4-Month-Old SAMP8 Mice Compared to SAMR1 Mice

**Fig. 2:**
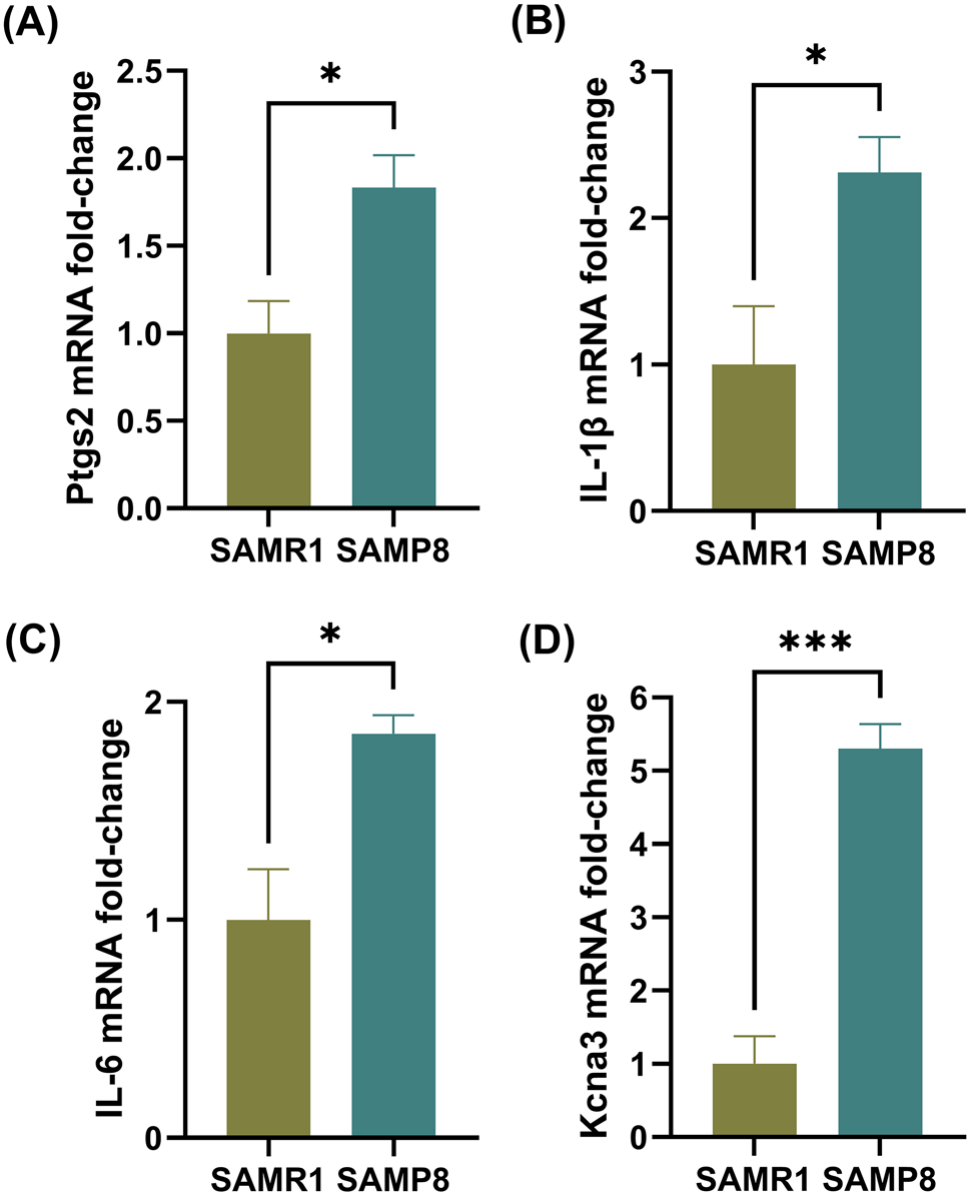
Relative mRNA expression of **A** Ptgs2, **B** IL-1β, **C** IL-6 and **D** Kcna3 in microglia isolated from 4-month-old SAMP8 mouse brains relative to those isolated from age-matched SAMR1 mouse brains. Data are presented as mean ± SEM (n = 3–4 mice per genotype); *p < 0.05 and ***p < 0.001 using a Student’s t-test

Relative to microglia from SAMR1 mice, the mRNA expression of Ptgs2 (a marker for pro-inflammatory microglia phenotype, Fig. 2A) and IL-1β and IL-6 (pro-inflammatory cytokines, Fig. 2B, C) were elevated in microglia from SAMP8 mice by 1.8-fold (p = 0.027), 2.3-fold (p = 0.031) and 1.8-fold (p = 0.012), respectively. The mRNA expression of Arg1 (a marker for anti-inflammatory microglia phenotype) was under the detection limit in both SAMR1 and SAMP8 microglia. In line with activation of microglia in SAMP8 mice, the mRNA expression of Kcna3 (Kv1.3) was 5.3-fold higher (p = 0.0004) in microglia from SAMP8 mice relative to microglia from SAMR1 mice (Fig. 2D). The pro-inflammatory activation of microglia and upregulation of Kv1.3 mRNA in the SAMP8 microglia provided a rationale to then assess the impact of microglial Kv1.3 blockade by chronic dosing of HsTX1[R14A] in 4-month-old SAMP8 mice.

### Chronic Dosing of HsTX1[R14A] Increased Brain Weight but did not Affect Body Weight of SAMP8 Mice

**Fig. 3:**
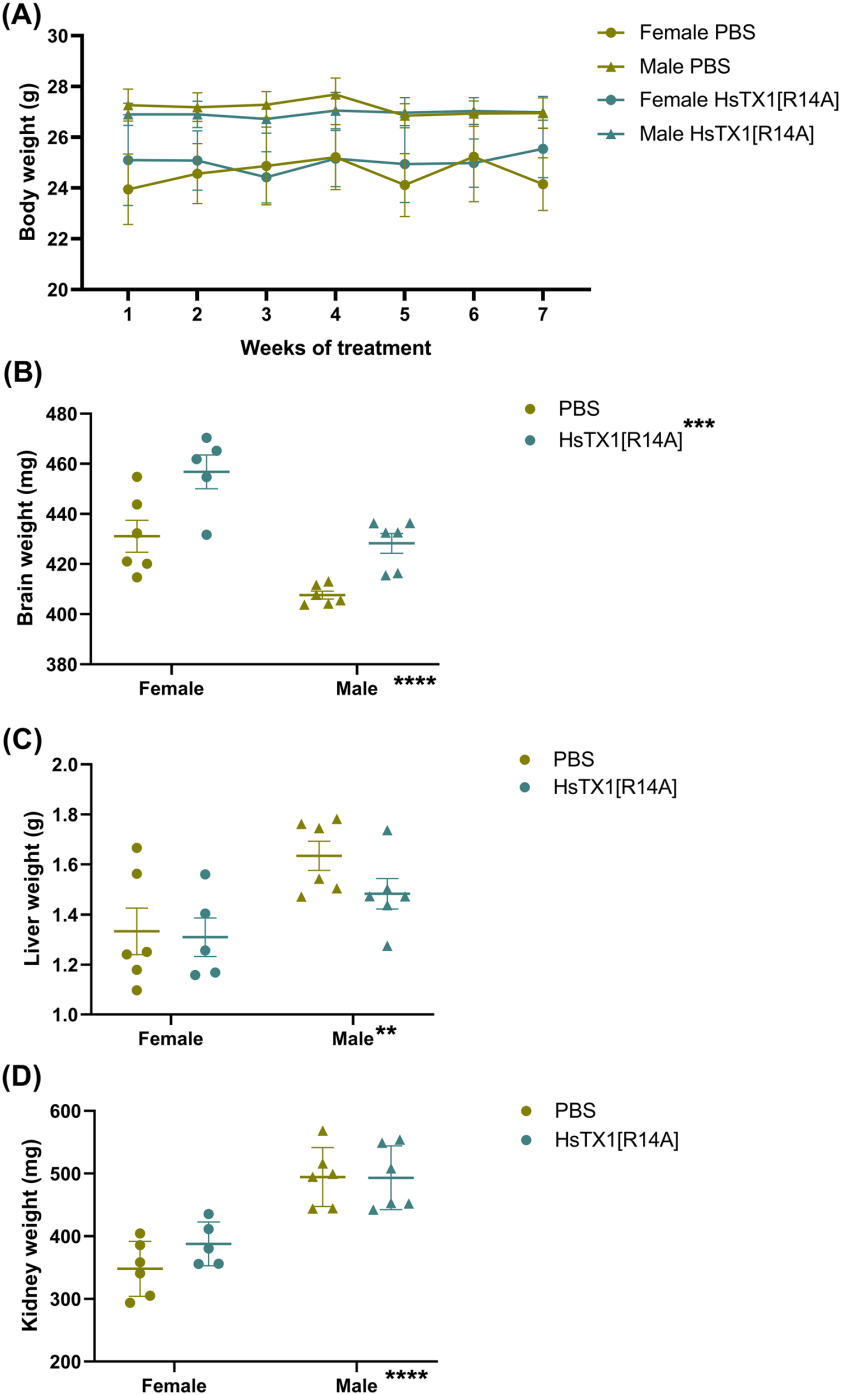
The weight of **A** body, **B** brain, **C** liver and **D** kidney of 6-month-old SAMP8 mice that had been administered subcutaneous HsTX1[R14A] (1 mg/kg) or PBS every other day for 2 months. Data are presented as mean ± SEM (n = 5–6 mice per group), with **p < 0.01, ***p < 0.001, ****p < 0.0001 using a two-way ANOVA

The body weight of mice was monitored weekly across the treatment, which was used to determine the injection volume of HsTX1[R14A] to ensure consistent inter-animal dosing. In addition, body weight is an important parameter to measure for behavioral assessment, as it may affect locomotor activity of mice and consequently, their performance in cognition assessment. A three-way ANOVA revealed a significant effect of gender (F_1,19_ = 7.89, p = 0.01) but no effect of HsTX1[R14A] treatment or duration of HsTX1[R14A] treatment on body weight (Fig. 3A), with the males having higher body weight than the females regardless of treatment. The brain weight was measured at the conclusion of treatment, to assess the macroscopic impact of HsTX1[R14A] on the brain. A two-way ANOVA indicated a significant effect of treatment (F_1,19_ = 21.71, p = 0.0002) and gender (F_1,19_ = 27.35, p < 0.0001) on brain weight (Fig. 3B). The brain weight of SAMP8 mice that were administered HsTX1[R14A] was greater than those that were administered PBS, and females had a greater brain weight than males. The weights of liver and kidneys were also assessed to check if HsTX1[R14A] enlarged/shrank these important peripheral organs. A two-way ANOVA only revealed a significant effect of gender, but not treatment, on the liver weight (F_1,19_ = 10.41, p = 0.004; Fig. 3C) and kidney weight (F_1,19_ = 44.83, p < 0.0001; Fig. 3D), with males having higher liver and kidney weights than females.

### HsTX1[R14A]-Treated SAMP8 Mice Performed Better than PBS-treated SAMP8 in T Maze Spontaneous Alternation 

**Fig. 4:**
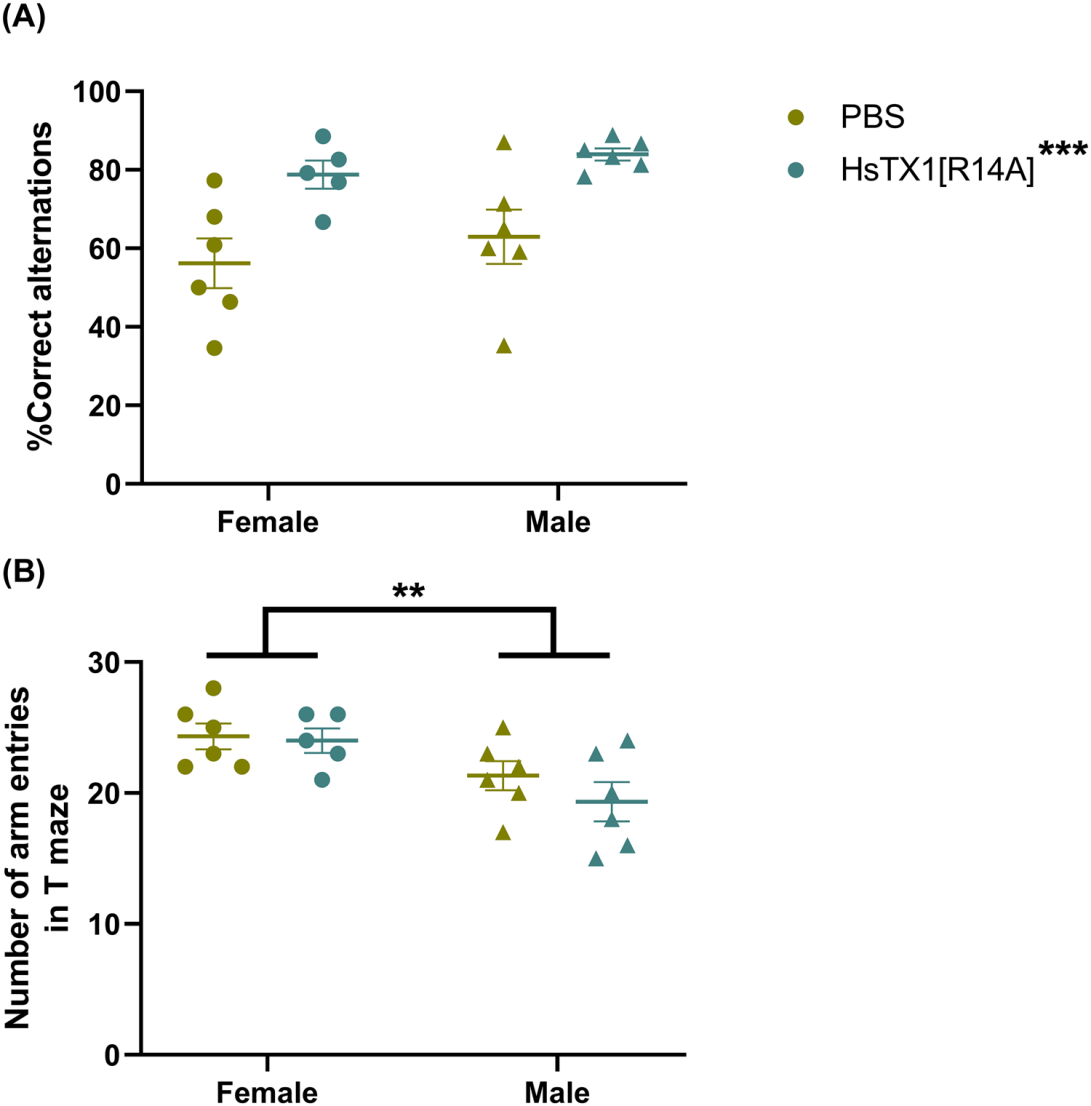
**A** The % of correct spontaneous alternations and **B** the number of arm entries in the T maze of SAMP8 mice that were administered every-other-day subcutaneous dosing of HsTX1[R14A] (1 mg/kg) or PBS. Data are presented as mean ± SEM (n = 5–6 mice per group), with **p < 0.01, and ***p < 0.001 (two-way ANOVA)

A two-way ANOVA revealed a significant main effect of HsTX1[R14A] treatment on spontaneous alternation (F_1,19_ = 17.25, p = 0.0005), but no effect of gender nor any interaction between treatment and gender (Fig. 4A). The % correct spontaneous alternation in T maze for SAMP8 mice receiving HsTX1[R14A] was significantly higher than that of the SAMP8 mice receiving PBS (81.58 ± 1.92% vs 59.58 ± 4.60%). The total number of arm entries was used to evaluate the locomotor activity of the mice. Although no significant effect of treatment was noted, a significant effect of gender was observed (F_1,19_ = 10.5, p = 0.0043), with males showing a modest reduction in the total number of arm entries compared to females (Fig. 4B).

### HsTX1[R14A]-Treated Female (but not Male) SAMP8 Mice Performed Better than PBS-Treated SAMP8 Mice in the Y Maze

**Fig. 5:**
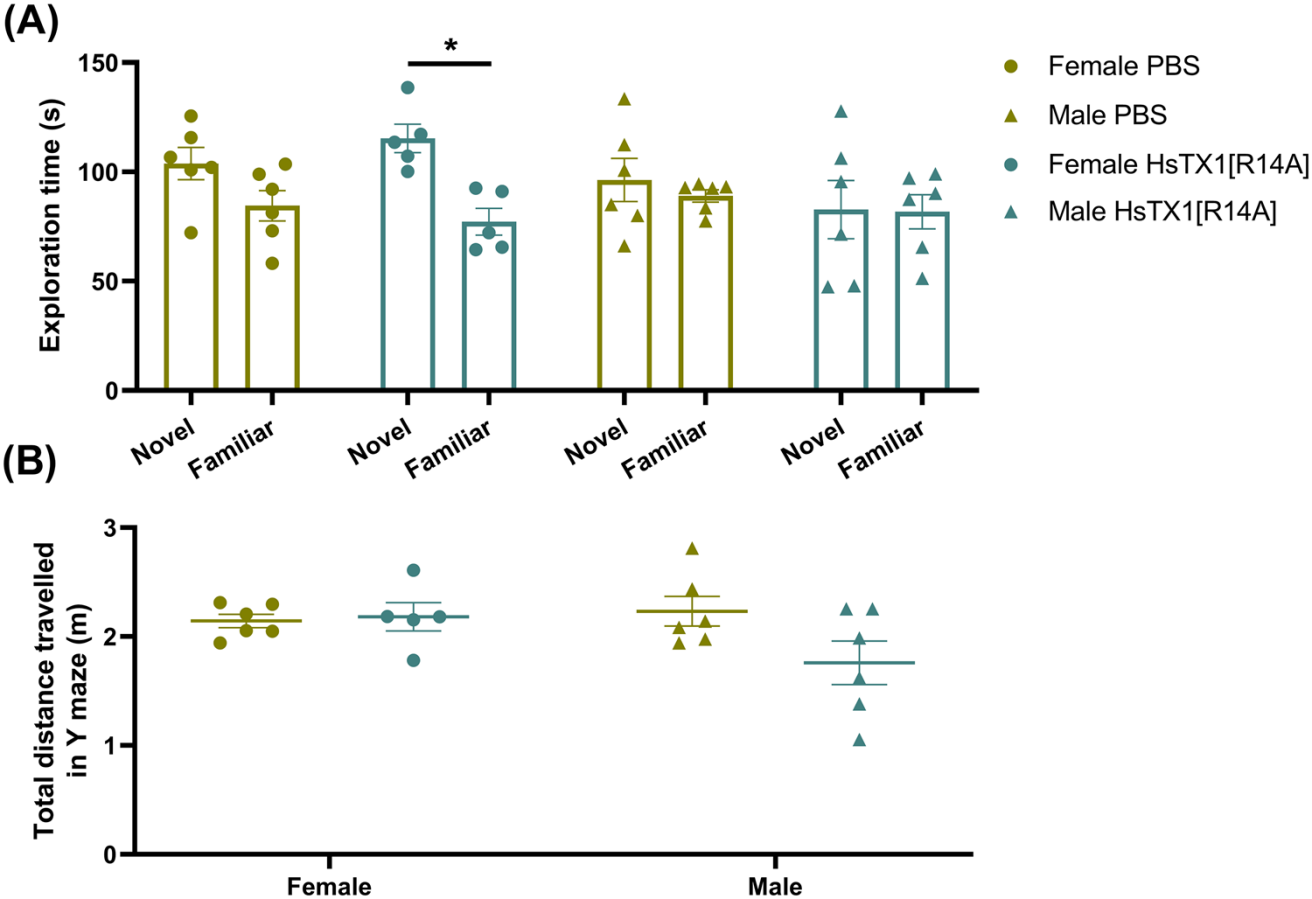
The performance of SAMP8 mice that were dosed subcutaneously with HsTX1[R14A] (1 mg/kg) or PBS every-other-day in the Y maze, showing **A** the time mice spent in the novel arm and familiar arm, and **B** the total distance travelled by the mice during the testing session. Data are presented as mean ± SEM (n = 5–6 mice per group), with *p < 0.05 (two-way ANOVA repeated measure, followed by post hoc pairwise comparison)

A three-way ANOVA revealed a significant effect of arm (F_1,19_ = 5.85, p = 0.03; Fig. 5A), and a trend for arm x gender interaction effect (F_1,19_ = 3.29, p = 0.08) in the Y maze. Post-hoc paired-wise comparison demonstrated that only female SAMP8 mice administered HsTX1[R14A] spent longer time in the novel arm than in the familiar arm (p = 0.02), suggesting an improvement in short-term spatial learning and memory. The total distance travelled by the mice in the Y maze was assessed, and no significant effect of treatment was observed (Fig. 5B). These data suggested that the altered arm exploration behavior was not due to altered locomotor activity of mice.

### HsTX1[R14A]-Treated SAMP8 Mice Performed Better than PBS-Treated SAMP8 Mice in the Novel Object Recognition Task

**Fig. 6:**
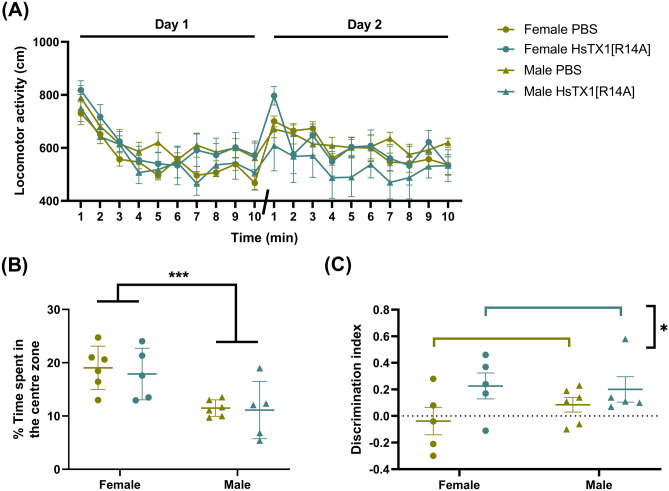
The NOR performance of SAMP8 mice that were administered subcutaneous HsTX1[R14A] (1 mg/kg) or PBS every-other-day, showing **A** the distance travelled by mice in the open arena over 10 min in the two habituation sessions on day 1 and day 2, **B** the % of time that mice spent in the centre zone during the day 1 habituation, and **C** the discrimination index of mice for object exploration in the NOR testing session. Data are presented as mean ± SEM (n = 5–6 mice per group), with *p < 0.05, and ***p < 0.001 (Two-way ANOVA)

The distance travelled by mice per min was recorded for the habituation sessions on day 1 and day 2 of the experiment (Fig. 6A). A three-way ANOVA revealed a significant effect of time (F_7.368,140_ = 15.49, p < 0.0001), suggesting that all mice, regardless of treatment or gender, were well acclimatized to the open arena. The time that mice spent in the centre of the arena during the day 1 acclimatization was used to evaluate anxiety levels in mice (Fig. 6B). Male SAMP8 mice spent a significantly lower proportion of exploration time in the centre of the arena compared to female SAMP8 mice, regardless of treatment (F_1,18_ = 16.54, p = 0.0007), indicating a higher anxiety level in male than in female mice. Of the 23 mice that received NOR training, two were removed due to object bias. For the testing session, a two-way ANOVA revealed a significant effect of treatment (F_1,17_ = 4.69, p = 0.04) but no effect of gender nor interaction between the two factors (Fig. 6C). It appeared that mice that received HsTX1[R14A] could differentiate the novel object from the familiar object while mice that received PBS failed to differentiate, as indicated by the discrimination index.

### HsTX1[R14A] Improved the Performance of Female SAMP8 Mice in the Water Maze

**Fig. 7:**
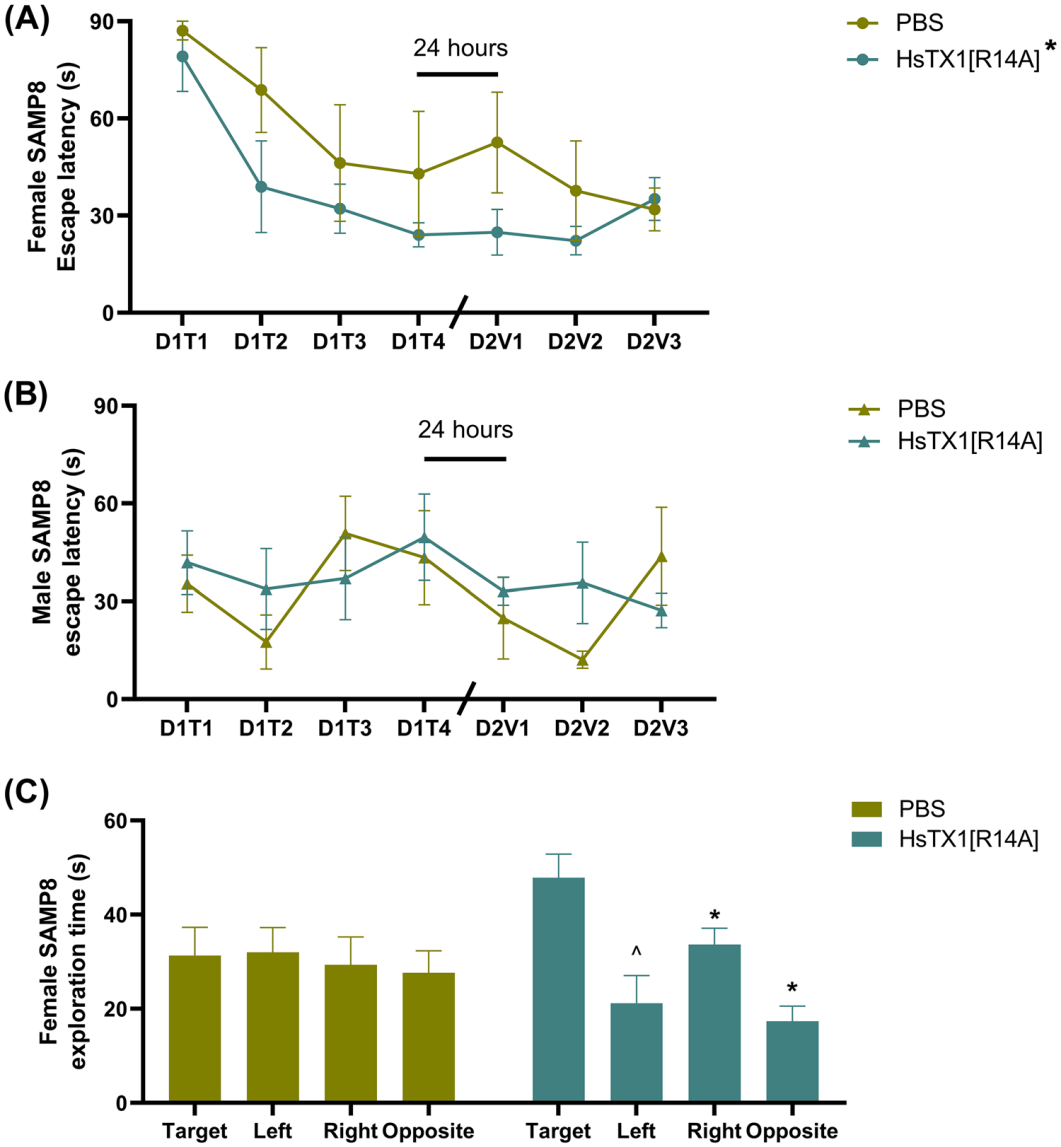
The water maze performance of SAMP8 mice that were administered subcutaneous HsTX1[R14A] (1 mg/kg) or PBS every-other-day, showing the escape platform visit latency of **A** the female and **B** the male mice in Day 1 training sessions (D1T1, D1T2, D1T3, D1T4) and Day 2 spatial trials (D2V1, D2V2, D2V3), and **C** the quadrant exploration time of mice on Day 3 probe trial. Data are presented as mean ± SEM (n = 4–6 mice per group), with *p < 0.05 and ^p < 0.1 (Two-way ANOVA)

A water maze was performed for both male and female SAMP8 mice that had been treated with HsTX1[R14A] or PBS. A learning curve was obtained for the female mice (Fig. 7A) but not for the male mice (Fig. 7B). This poor performance of male SAMP8 mice in the water maze could possibly be due to elevated stress/anxiety [44], which are more apparent in male SAMP8 mice as per a previous report [45] and as demonstrated by our study (Fig. 6B). We therefore only performed statistical analysis for female SAMP8 mice, from which the mixed-effect analysis revealed a significant effect of training (F_3.249,22.74_ = 4.59, p = 0.01) and treatment (F_1,8_ = 5.78, p = 0.04). The learning curves showed that that female SAMP8 mice treated with HsTX1[R14A] required less training than those dosed with PBS in order to exhibit a comparable latency to locate the escape platform. No significant difference between D2V1 and D1T4 were noted in female SAMP8 mice regardless of treatment, which indicated intact long-term spatial memory in these mice. However, a high variability was noted in female SAMP8 mice that were administered PBS, which could reduce the sensitivity of the test. For the probe trial (Fig. 7C), a two-way ANOVA revealed a trend for a main effect of quadrant (F_2.268,18.14_ = 2.704, p = 0.08). Post-hoc analysis demonstrated that the time the HsTX1[R14A]-treated female SAMP8 mice spent in the target quadrant was significantly longer than that in the right and opposite quadrant (p = 0.04 and p = 0.02, respectively) and had a trend to be more than that in the left quadrant (p = 0.08). No significant difference in the time spent in different quadrants was noted in female SAMP8 mice administered PBS.

### HsTX1[R14A] Alters the mRNA Profile of Key Disease-Modifying Genes in Mouse Brain

**Fig. 8:**
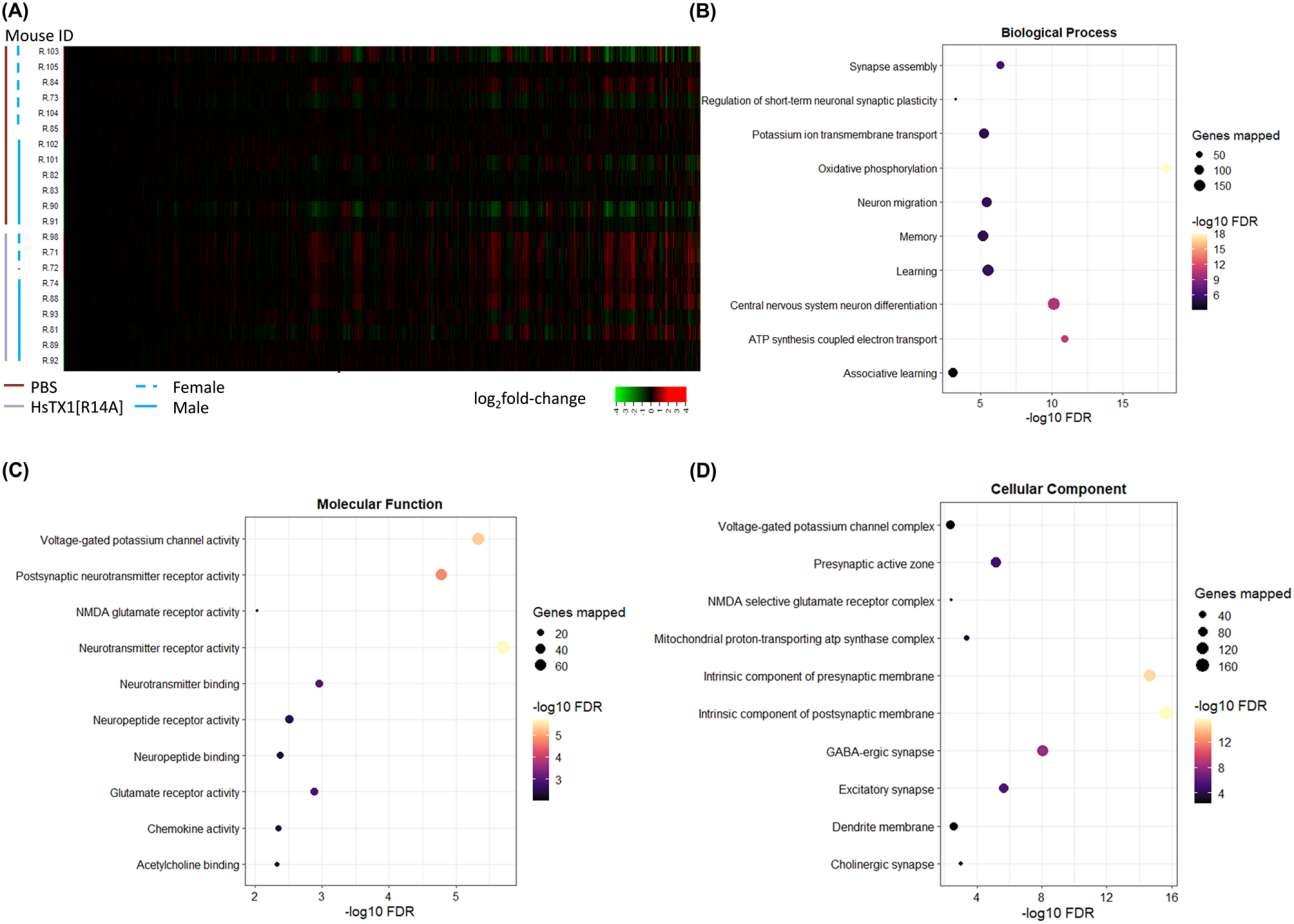
The transcriptomic profiling of brains isolated from 6-month-old SAMP8 mice that had been administered subcutaneous HsTX1[R14A] (1 mg/kg) or PBS for 2 months every other day (n = 9–12 mice per genotype), showing **A** heat map of all genes detected, and dot plots for functional enrichment of **B** biological process, **C** molecular function, and **D** cellular component

**Fig. 9:**
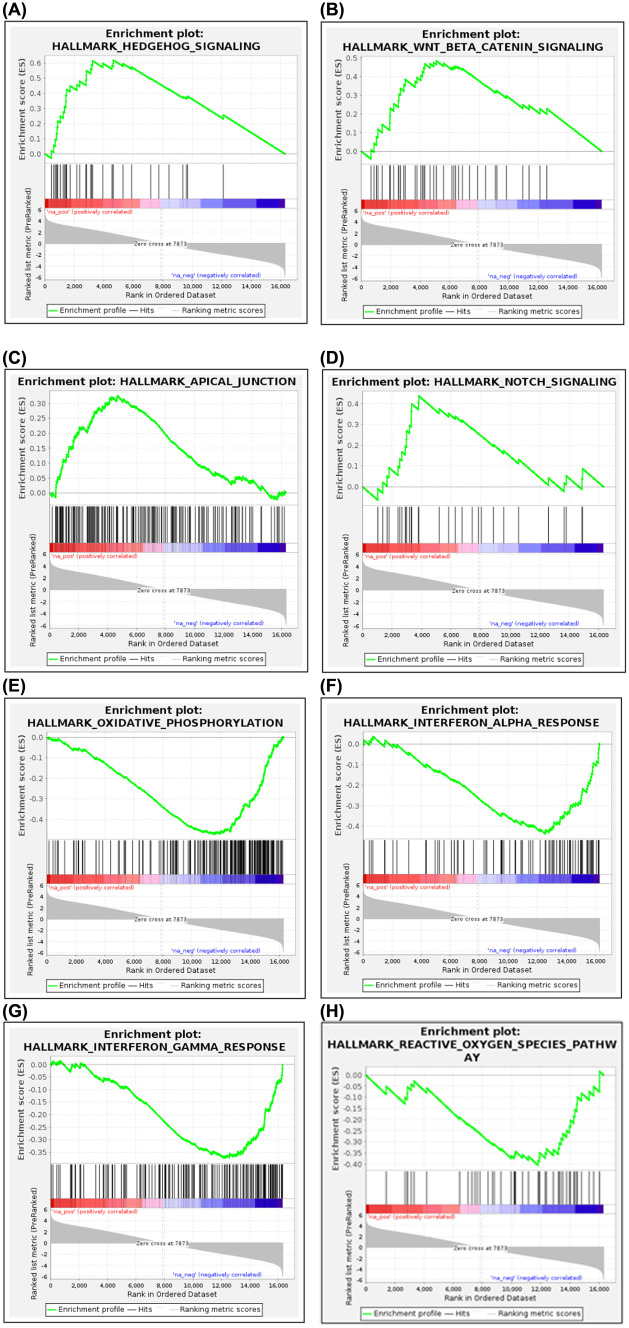
GSEA enrichment plots for hallmark gene sets, showing the upregulated gene sets **A** Hedgehog signaling, **B** Wnt/beta-catenin signaling, **C** apical junction, **D** notch signaling, and the downregulated gene sets **E** oxidative phosphorylation, **F** interferon alpha response, **G** interferon gamma response, **H** reactive oxygen species pathway, in brains from 6-month-old SAMP8 mice that had been treated with HsTX1[R14A] (n = 9–12 mice per genotype)

**Fig. 10:**
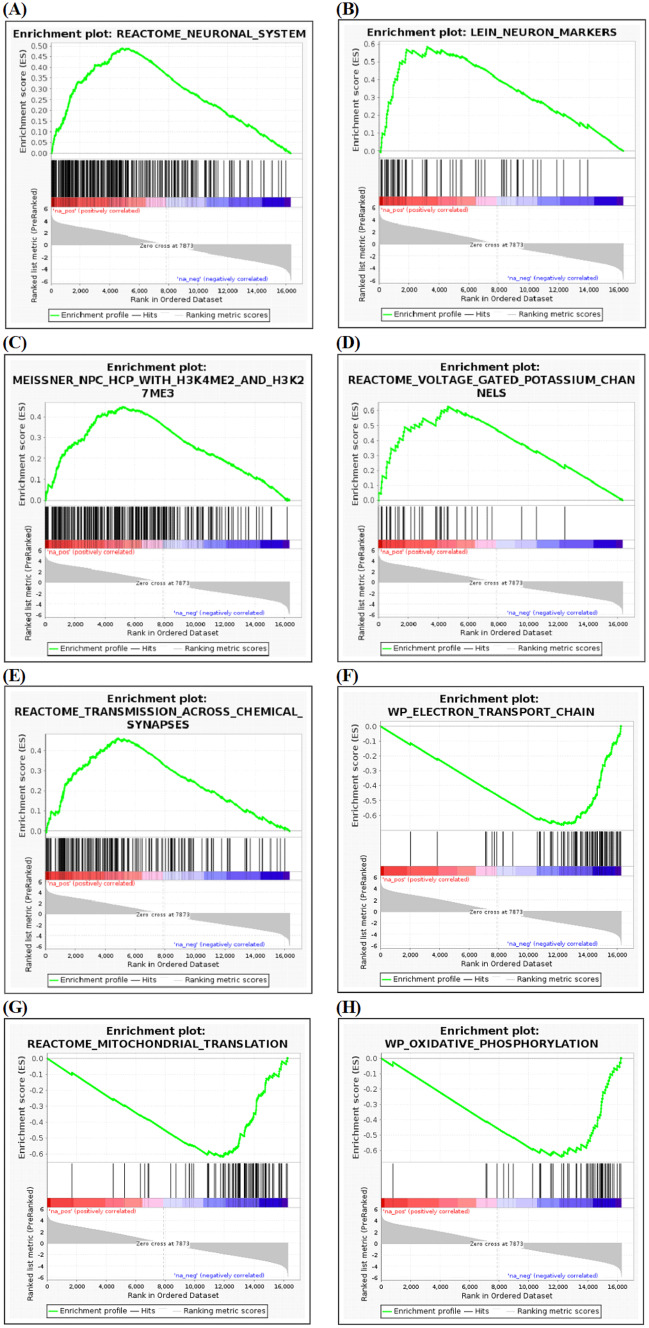
GSEA enrichment plots for curated gene sets, showing the upregulated gene sets **A** Reactome neuronal system, **B** LEIN neuron markers, **C** MEISSNER NPC HCP with H3K4ME2 and H3K27ME3, **D** Reactome voltage gated potassium chanels, **E** Reactome transmission across chemical synapses, and the downregulated gene sets **F** WP electron transport chain, **G** Reactome mitochondrial translation, and **H** WP oxidative phosphorylation, in brains from 6-month-old SAMP8 mice that had been treated with HsTX1[R14A] (n = 9–12 mice per genotype)

To explore the impact of HsTX1[R14A] on global processes in the brain, RNA-sequencing analysis was performed. Differential expression gene analysis revealed that, of the 16318 genes detected, 5760 genes were statistically significantly-differentially expressed (FDR $\le$ 0.05) between HsTX1[R14A]-treated and PBS-treated mouse brains; of these, 60 were upregulated greater than twofold (log_2_FC ≥ 1) and 30 were downregulated greater than twofold (log_2_FC $\le$ 1) (Fig. 8A). The gene expression levels are available in Supplementary file 1. Functional enrichment analysis was also performed; the biological process, molecular function and cellular component highlighted by the analysis are graphically presented in Fig. 8B-D, with the complete list available in Supplementary file 2. GSEA was performed on pre-ranked data (using limma t.stat function) comparing brains from PBS-treated vs HsTX1[R14A]-treated SAMP8 mice. For the 50 hallmark gene sets, 24 gene sets were upregulated (with 10 gene sets having FDR q-val < 0.05) and 26 gene sets were downregulated (with 12 gene sets having FDR q-val < 0.05) in brains from HsTX1[R14A]-treated mice. A total of 1584 m2 curated gene sets were used for analysis, of which 819 were upregulated (with 229 gene sets having FDR q-val < 0.05) and 765 were downregulated (with 241 gene sets having FDR q-val < 0.05). Within the top 20 gene sets with the highest/lowest normalized enrichment score, the enrichment plots that are most relevant to the cognitive beneficial effect of HsTX1[R14A] are presented in Fig. 9 (hallmark gene sets) and Fig. 10 (m2 curated gene sets). The complete GSEA results are available in Supplementary file 3.

## Discussion

The voltage-gated potassium channel Kv1.3 is a target of interest in the development of novel therapeutics for neurodegenerative diseases such as AD. Our previous studies, together with those of others [23, 46], provide evidence that microglial Kv1.3 is involved in the production of pro-inflammatory mediators, and that pharmacological blockade or genetic deletion of microglial Kv1.3 substantially reduces microglial pro-inflammatory responses. Thus, there is clear evidence that Kv1.3 is a promising therapeutic target to modulate microglia-mediated neuroinflammation in neurodegenerative diseases. However, a potential impact on non-microglial cell types cannot be discounted, as it has also been demonstrated that genetic silencing or pharmacological blockade of Kv1.3 promotes neuronal survival [17] and neurogenesis [16]. We build on these studies by assessing the therapeutic potential of a very selective and potent peptide-based Kv1.3 blocker, HsTX1[R14A].

In this study, microglia isolated from 4-month-old SAMP8 mice exhibited a stronger pro-inflammatory activation phenotype than microglia from SAMR1 mice, alongside a substantial increase in Kv1.3 expression. SAMR1 mice are routinely used as a control for SAMP8 mice [47, 48], given that they are on the AKR/J background, as are the SAMP8 mice [49]. However, SAMP8 mice are bred separately from SAMR1 mice to maintain the AD-like phenotype, and therefore the SAMR1 and SAMP8 mice are considered as separate strains. There is no current “wildtype” littermate control for SAMP8 mice, given that the sporadic mutation only occurs within SAMP8 mice. While the SAMR1 mice are still routinely used as a control for SAMP8 mice by many researchers [47, 48, 50], some researchers perform comparative studies between young and old SAMP8 mice [51], in an attempt to identify disease progression-related changes, but this would be comparing not only disease progression but also age. Given the elevation of microglial Kv1.3 expression in the 4-month-old SAMP8 mice, we evaluated the effects of HsTX1[R14], a specific Kv1.3 blocker, on cognitive function and brain transcriptomic profiling in SAMP8 mice. We observed that HsTX1[R14A] increased the brain weight of SAMP8 mice, regardless of gender. This is possibly due to a reduction in neuroinflammation from microglial Kv1.3 blockade. In fact, it has been reported previously that therapeutics that suppress neuroinflammation can prevent brain atrophy [52]. The increased brain weight in HsTX1[R14A]-treated mice is unlikely to be due to brain edema as an adverse effect, given that ShK-170 (another Kv1.3 blocker) has been reported to reduce brain edema [53].

SAMP8 mice treated with HsTX1[R14A] exhibited improved working memory compared to SAMP8 mice treated with PBS, as soon as after 4 weeks of dosing, as demonstrated in the T maze spontaneous alternation test. In addition, HsTX1[R14A] improved the performance of female SAMP8 mice in the Y maze short-term spatial learning and memory test. The lack of cognitive-protective effects of HsTX1[R14A] in male SAMP8 mice in the Y maze could be due to the observation that the male mice exhibited more robust cognitive impairment than age-matched female mice, as has been reported previously [54]. In fact, we observed that the brain weight of male SAMP8 mice was significantly lower than that of the female mice, which could also be attributed to more severe/gender-specific pathology. In future studies, HsTX1[R14A] treatment could be trialed in younger male SAMP8 mice, where effects on short-term spatial learning and memory might be observed. Nevertheless, we demonstrated that associative memory in SAMP8 mice can be improved by HsTX1[R14A] treatment, as demonstrated in the NOR task, regardless of gender. The lack of a gender-specific effect of HsTX1[R14A] in the NOR task could be attributed to the fact that the brain regions responsible for short-term spatial memory and association memory are different, and the different extent of damage to relevant brain regions in SAMP8 mice [55].

HsTX1[R14A] improved spatial learning in female SAMP8 mice in the 3-day water maze test. We did not observe a proper learning curve for the male SAMP8 mice using this protocol regardless of treatment, which could partly be due to anxiety, as male SAMP8 mice exhibit greater anxiety-like behavior than the female SAMP8 mice (Fig. 6B). However, an alternative explanation is that HsTX1[R14A] could not rescue the impaired spatial learning and memory in male SAMP8 mice, in agreement with their performance in the Y maze. On the other hand, the difference between D2V1 and D1T4 clearly showed that female SAMP8 mice treated with HsTX1[R14A] had intact long-term spatial memory. However, for PBS-treated SAMP8 mice, there was a great variability in their performance in the water maze for D1T4 and D2V1, which could result in our test being less sensitive in detecting long-term spatial memory deficits in the PBS-treated female SAMP8 mice. In addition, HsTX1[R14A]-treated female SAMP8 mice exhibited intact memory retrieval, as they spent significantly more time in the target quadrant (where the escape platform was) relative to other quadrants. However, this pattern was not observed in PBS-treated SAMP8 mice. Overall, the water maze data suggested that HsTX1[R14A] could rescue spatial learning and memory and memory retrieval in female SAMP8 mice.

Given that the behavioral assessments strongly supported a cognitive-enhancing effect of HsTX1[R14A] in SAMP8 mice, especially females, we performed transcriptomic profiling of the brains to assess the impact of HsTX1[R14A] on the whole brain. This is to explore biological processes, molecular function, cellular components and pathways in the brain (not limited to microglia) that were affected by HsTX1[R14A]. Both functional enrichment analysis and GSEA indicated that genes associated with voltage-gated potassium channel activity are upregulated. This could be a compensatory response to the blockade of Kv1.3 by HsTX1[R14A]. We also attempted to assess cytokine levels (TNF-α, IL-6, IL-1β) in the brain using ELISA, but they were below the detection limit (data not shown). These cytokines were previously reported at < 1 pg/mg in 6-month-old SAMP8 mice [56]. Despite the lack of cytokine data, the modulatory effect of HsTX1[R14A] on microglia-mediated neuroinflammation was identified in the GSEA analysis. For example, genes involved in Notch signaling (an important pathway in regulating inflammation in the CNS) are upregulated in brains from HsTX1[R14A]-treated SAMP8 mice compared to brains from PBS-treated SAMP8 mice. It has been reported that the activation of Notch signaling in microglia induces a decrease in the release of pro-inflammatory cytokines and nitric oxide production, as well as an increase in phagocytosis [57]. This is in line with the increase in microglial phagocytosis reported in APP/PS1 mice that were administered PAP-1 (a non-specific Kv1.3 blocker) [12]. From as early as 6 months of age, SAMP8 mice exhibit Aβ deposition in the hippocampus that increases with age [58]. Whether HsTX1[R14A] may improve the microglial phagocytosis of Aβ to attenuate Aβ deposition in the hippocampus of SAMP8 mice, as has been suggested with non-selective Kv1.3 blockade in APP/PS1 mice [12], could be assessed in further studies. On the other hand, genes involved in Hedgehog signaling (a pathway involved in regulating pathogenic inflammation within the CNS) were upregulated. As pharmacological activation of the Hedgehog pathway can counteract ongoing excessive CNS inflammation and effectively halt disease progression [59], this upregulated gene set also implied that HsTX1[R14A] can effectively suppress microglia-mediated neuroinflammation. In addition, GSEA also highlighted downregulation by HsTX1[R14A] of gene sets for the interferon-α response [60], interferon-γ response [61], and reactive oxygen species pathway [62], all of which are related to microglia activity and have regulatory roles in CNS inflammation. Interestingly, functional enrichment analysis and GSEA indicated a downregulation of genes associated with oxidative phosphorylation, electron transport chain, and mitochondrial translation in brains from HsTX1[R14A]-treated mice. It is known that activated astrocytes and microglia (e.g. during neuroinflammation) have increased energy demand that is met by upregulation of mitochondrial function [63, 64]. The downregulation of these gene sets implies that HsTX1[R14A] effectively suppresses microglial activation in SAMP8 mice.

It has to be emphasized that Kv1.3 channels are expressed in several subsets of neurons and in all types of glial cells (astrocytes, oligodendrocytes, and microglia) [16], and neural progenitor cells [18]. Therefore, the cognitive benefits mediated by HsTX1[R14A] may also be due to the modulation of non-microglia cells. For example, blockade of Kv1.3 channels has been reported to promote neural progenitor cell proliferation, differentiation and maturation [18, 65]. The Wnt/β-catenin signaling pathway is a significant pathway regulating cell proliferation, migration and differentiation, driving adult stem cells in mammals; impaired Wnt/β-catenin signaling possibly plays an important role in the pathogenesis of AD [66]. Our GSEA indicated that genes associated with Wnt/β-catenin signaling are upregulated, which implies a possible impact of HsTX1[R14A] on neurogenesis [67] in SAMP8 mice. This is further supported by our functional enrichment analysis, which highlighted the upregulation of genes associated with central nervous system neuron differentiation (GO:0021953).

A recent study has demonstrated that blocking Kv1.3 in 5 × FAD mice (a model of familial AD) increases synaptic protein expression. Our functional enrichment analysis and GSEA agree with this finding, where genes associated with synapse assembly (GO:0007416, and Reactome transmission across chemical synapses) are upregulated in brains from HsTX1[R14A]-treated mice. In addition, GSEA also highlighted an upregulation in Reactome neuronal system and LEIN neuron markers, and the functional enrichment analysis revealed that genes associated with learning (GO:0007612) and memory (GO:0007613) are upregulated in brains from HsTx1[R14A]-treated SAMP8 mice. However, whether these effects are a direct consequence of Kv1.3 blockade or a subsequent effect from reduced neuroinflammation is yet to be investigated. Overall, the transcriptomic profiling data highlighted pathways that were consistent with the cognitive benefit observed in the behavioral studies. However, it must be emphasized that further experiments are required to obtain mechanistic insights into how HsTX1[R14A] interferes with the pathways highlighted by functional enrichment analysis and GSEA in different cell types.

This study demonstrates that HsTX1[R14A] improves cognitive function in a mouse model of sporadic AD and provides proof-of-concept results to support the use of Kv1.3 blockers as potential therapeutics for AD. The beneficial effects of HsTX1[R14A] are associated with transcriptomic demonstration of reduced neuroinflammation and improved synaptic function and neurogenesis, confirming the neuroinflammation-attenuating effects of HsTX1[R14A] in AD.

## Supplementary Information

Below is the link to the electronic supplementary material.

Supplementary file1 (XLS 6006 KB)

Supplementary file2 (XLS 1488 KB)

Supplementary file3 (XLS 407 KB)

## Data Availability

The data supporting the findings of this study are available from the corresponding authors, upon reasonable request.
